# Modifications of Sarcoplasmic Reticulum Function Prevent Progression of Sarcomere-Linked Hypertrophic Cardiomyopathy Despite a Persistent Increase in Myofilament Calcium Response

**DOI:** 10.3389/fphys.2020.00107

**Published:** 2020-03-10

**Authors:** Shamim A. K. Chowdhury, Chad M. Warren, Jillian N. Simon, David M. Ryba, Ashley Batra, Peter Varga, Evangelia G. Kranias, Jil C. Tardiff, R. John Solaro, Beata M. Wolska

**Affiliations:** ^1^Department of Physiology and Biophysics and the Center for Cardiovascular Research, College of Medicine, University of Illinois at Chicago, Chicago, IL, United States; ^2^Department of Pediatrics, Section of Cardiology, University of Illinois at Chicago, Chicago, IL, United States; ^3^Department of Pharmacology and Systems Physiology, University of Cincinnati, Cincinnati, OH, United States; ^4^Department of Medicine, Division of Cardiology, The University of Arizona, Tucson, AZ, United States; ^5^Department of Medicine, Division of Cardiology, University of Illinois at Chicago, Chicago, IL, United States

**Keywords:** hypertrophic cardiomyopathy, Ca^2+^/calmodulin-dependent protein kinase II (CaMKII), myofilament Ca^2+^ sensitivity, phospholamban, troponin T (TnT), speckle strain, treatment

## Abstract

Hypertrophic cardiomyopathy (HCM) is a genetic disorder caused by mutations in different genes mainly encoding myofilament proteins and therefore called a “disease of the sarcomere.” Despite the discovery of sarcomere protein mutations linked to HCM almost 30 years ago, the cellular mechanisms responsible for the development of this disease are not completely understood and likely vary among different mutations. Moreover, despite many efforts to develop effective treatments for HCM, these have largely been unsuccessful, and more studies are needed to better understand the cellular mechanisms of the disease. In experiments reported here, we investigated a mouse model expressing the mutant cTnT-R92Q, which is linked to HCM and induces an increase in myofilament Ca^2+^ sensitivity and diastolic dysfunction. We found that early correction of the diastolic dysfunction by phospholamban knockout (PLNKO) was able to prevent the development of the HCM phenotype in troponin T (TnT)-R92Q transgenic (TG) mice. Four groups of mice in FVB/N background were generated and used for the experiments: (1) non-transgenic (NTG)/PLN mice, which express wild-type TnT and normal level of PLN; (2) NTG/PLNKO mice, which express wild-type TnT and no PLN; (3) TG/PLN mice, which express TnT-R92Q and normal level of PLN; (4) TG/PLNKO mice, which express TnT-R92Q and no PLN. Cardiac function was determined using both standard echocardiographic parameters and speckle tracking strain measurements. We found that both atrial morphology and diastolic function were altered in TG/PLN mice but normal in TG/PLNKO mice. Histological analysis showed a disarray of myocytes and increased collagen deposition only in TG/PLN hearts. We also observed increased Ca^2+^/calmodulin-dependent protein kinase II (CaMKII) phosphorylation only in TG/PLN hearts but not in TG/PLNKO hearts. The rescue of the HCM phenotype was not associated with differences in myofilament Ca^2+^ sensitivity between TG/PLN and TG/PLNKO mice. Moreover, compared to standard systolic echo parameters, such as ejection fraction (EF), speckle strain measurements provided a more sensitive approach to detect early systolic dysfunction in TG/PLN mice. In summary, our results indicate that targeting diastolic dysfunction through altering Ca^2+^ fluxes with no change in myofilament response to Ca^2+^ was able to prevent the development of the HCM phenotype and should be considered as a potential additional treatment for HCM patients.

## Introduction

Hypertrophic cardiomyopathy (HCM) is a genetic disorder caused by mutations in different genes mainly encoding myofilament proteins and therefore called a “disease of the sarcomere” (Ashrafian et al., 2011; Towbin, 2014; Marian and Braunwald, 2017). It is characterized by early onset of diastolic dysfunction, myocyte disarray, and a variable degree of fibrosis and progressive hypertrophy and later onset of systolic dysfunction. Symmetrical left ventricular or asymmetrical septal hypertrophy typifies the disorder, but some mutations do not result in thickening of left ventricle (LV) walls. HCM is the leading cause of sudden cardiac death (SCD) in young people typically associated with exercise (Maron and Maron, 2016). Despite the discovery of sarcomere protein mutations linked to HCM almost 30 years ago (Geisterfer-Lowrance et al., 1990), the cellular mechanisms responsible for the development of this disease are not completely understood and likely vary among different mutations. Moreover, despite multiple studies to discover specific treatments (Marian, 2009; Alves et al., 2010; Ashrafian et al., 2011; Tardiff et al., 2015), there are no specific treatments for HCM patients. Patients are treated for symptoms of left ventricular outflow tract (LVOT) obstruction or decreased cardiac output with negative inotropic medications, and the common complication of arrhythmias is treated both pharmacologically and with implanted defibrillators. Septal reduction by surgery and ethanol (EtOH) ablation procedures are often performed to alleviate LVOT obstruction (Gersh et al., 2011; Marian and Braunwald, 2017).

Initial diastolic dysfunction in HCM patients is triggered by altered myofilament properties induced by protein mutations. However, the overall HCM phenotype depends not only on the mutation *per se* but also on additional contributing factors during progression of the disease (Arad et al., 2002; Deranek et al., 2019). These factors include both the response of myofilament force and kinetics to Ca^2+^ as well as membrane-controlled Ca^2+^ fluxes to and from the myofilaments. In experiments reported here, we investigated a mouse model expressing mutant cTnT-R92Q, which induces an increase in myofilament Ca^2+^ sensitivity and results in diastolic dysfunction. We tested whether prevention of the diastolic dysfunction is able to delay or stop the development of HCM phenotype in troponin T (TnT)-R92Q mice. Our approach was to promote sarcoplasmic reticulum (SR) Ca^2+^ uptake in the TnT-R92Q HCM mouse model by phospholamban knockout (PLNKO). We determined cardiac function (using both standard echocardiographic parameters and speckle strain measurements), histology, and myofilament function. We also established levels of Ca^2+^/calmodulin-dependent protein kinase II (CaMKII) phosphorylation, which has been reported to be a key signaling pathway in human HCM (Helms et al., 2016) and in mouse HCM models (Lehman et al., 2019). We observed that KO of PLN in hearts of cTnT-R92Q mice resulted in decreased CaMKII phosphorylation and a prevention of the development of the HCM phenotype, although the increase in myofilament Ca^2+^ sensitivity was preserved.

## Materials and Methods

### Generation of New Transgenic Mice

Transgenic (TG) TnT-R92Q heterozygous mice with Myc-tag at NH_2_-termini were originally generated and characterized in C57BL/6 genetic background (Tardiff et al., 1999). PLNKO mice used in this project were in FVB/N genetic background (Gaffin et al., 2011). Since it is well-documented that genetic background may have a significant effect on the HCM phenotype (Prabhakar et al., 2001; Michele et al., 2002; Rowlands et al., 2017), we first rederived and characterized TnT-R92Q mice in FVB/N background. We crossed TnT-R92Q heterozygous male with FVB/N female and created F1 generation of mice. Next, we bred TnT-R92Q-positive male from F1 generation with FVB/N female to obtain F2 generation of mice. We repeated this process up to F10 generation. To generate PLNKO mice expressing mutated TnT-R92Q, we bred positive male from F10 generation with PLNKO female. All mice from this breeding were heterozygous for PLN and express either normal wild-type TnT or mutated TnT (TnT-R92Q). Next, we bred heterozygous PLN male mouse that was positive for TnT-R92Q transgene with PLNKO female. This breeding allowed us to generate PLNKO mice that also expressed TnT-R92Q. These mice were used for further breeding with PLNKO females. The following abbreviations for the four groups of mice in FVB/N background were used: (1) non-transgenic (NTG)/PLN mice, which express wild-type TnT and normal level of PLN; (2) NTG/PLNKO mice, which express wild-type TnT and no PLN; (3) TG/PLN mice, which express TnT-R92Q and normal level of PLN; (4) TG/PLNKO mice, which express TnT-R92Q and no PLN. All measurements in our studies were done at 16 weeks of age.

### High-Resolution Echocardiography

Mice were initially anesthetized in a plexiglass chamber connected to a vaporizer providing isoflurane at 3% in 100% O_2_ (Alves et al., 2014). Once induced, the mouse was secured in the supine position on a warming plate, and the isoflurane concentration was reduced to 1.5–2%, and hair was removed from the chest using a depilating agent. Body temperature was monitored and kept close to 37°C throughout the procedure. Transthoracic echocardiography was performed using a Vevo 770 High-Resolution *In Vivo* Imaging System and scan head with a center frequency of 40 MHz (VisualSonics, Toronto, ON, Canada). Anatomical M-Mode images of the left ventricle (LV) aortic root and left atrium (LA) were taken from the parasternal long axis view. The parasternal short axis view at the level of the papillary muscles was used to measure the LV internal dimension, anterior and posterior wall thicknesses. Pulsed-wave Doppler was performed in the apical four-chamber view. The mitral inflow was recorded with the Doppler sample volume at the tips of the mitral valve leaflets to obtain the peak velocities of flow in the early phase of diastole (E) and after LA contraction (A). The Doppler sample volume was moved toward the left ventricular outflow tract (LVOT), and both the mitral inflow and LV outflow were simultaneously recorded to measure the isovolumic relaxation time. Additional information about diastolic function was obtained with tissue Doppler imaging. Peak diastolic myocardial velocities in the early phase of diastole (e′) and after LA contraction (a′) and systolic velocity (s′) were obtained with the sample volume at the septal side of the mitral annulus in the four-chamber view. All measurements and calculations were averaged from three consecutive cycles and performed according to the American Society of Echocardiography guidelines. Data analysis was performed off-line with the Vevo 770 Analytic Software (VisualSonics, Toronto, ON, Canada). In addition, to generate data used for strain measurements, one cohort of mice was scanned using a Vevo 2100 High-Resolution *In Vivo* Imaging System and RMVTM MS-550D scan head with a center frequency of 40 MHz (VisualSonics, Toronto, ON, Canada), and images in short and long axes were obtained as described above. VevoStrain software was used to analyze the data (Bauer et al., 2011). Four to five consecutive cardiac cycles were selected for the analysis, and semiautomated tracing of the endocardial and epicardial borders was used. The tracing was corrected as needed, and longitudinal (LS), circumferential (CS), and radial strain (RS) and strain rates were calculated.

### Force-Ca^2+^ Relations of Detergent-Extracted Myofiber Bundles

Force-Ca^2+^ relation measurements were performed as previously described (Ryba et al., 2019). Mice were heparinized (1,000 IU/kg) and anesthetized using 200 mg ketamine/20 mg xylazine/kg body weight. All mice were euthanized by cardiectomy under surgical anesthesia in accordance with the American Veterinary Medical Association Panel on Euthanasia Guidelines (American Veterinary Medical Association, 2013). Left ventricular papillary muscles were then isolated, dissected into fiber bundles approximately 200 μm in width and 3–4 mm in length, and detergent-extracted in a high relaxing (HR) solution (10 mmol/L EGTA, 41.89 mmol/L K-Propinate, 100 mmol/L BES, 6.75 mmol/L MgCl_2_, 6.22 mmol/L Na_2_ATP, 10 mM Na_2_CrP, 5 mmol/L NaN_3_, pH 7.0) with 1% v/v Triton X-100 for 3–4 h at 4°C. The HR solution was then replaced with HR solution without Triton X-100. Free Ca^2+^ concentrations were calculated using WEBMAXC STANDARD and ranged from pCa (−log [Ca^2+^]) values of 8.0 to 4.5. Free Ca^2+^ concentrations were generated by mixing varying ratios of HR solution with solution containing 10 mmol/L CaCl_2_. Fiber bundles were mounted between a micromanipulator and a force transducer and bathed in HR solution. We measured sarcomere length, which was kept constant throughout the experiment at 2.2 μm, using a He-Ne laser diffraction pattern to make adjustments with a micromanipulator. The fibers were initially stimulated to generate force at pCa 4.5 and placed back into the HR solution, and the width and diameter were measured along three points. Fibers were then subjected to sequential increases in Ca^2+^ concentration; their developed force was recorded on a chart recorder. All experiments were carried out at 23°C.

### Histology

Histology was performed as previously described (Gaffin et al., 2011) with some slight modifications. Mice were anesthetized using 200 mg ketamine/20 mg xylazine per kg body weight. The hearts were quickly removed and retrogradely perfused through the aorta with ice-cold saline followed by 10% formalin. The heart was then transversely sliced into four pieces, and each piece was placed into a cassette. Cassettes were kept in formalin and shipped to the Veterinary Diagnostic Laboratory at the University of Illinois at Urbana-Champaign (UIUC) College of Veterinary Medicine for paraffin embedding and sectioning. Sections (5 μm) were placed on slides and stained using hematoxylin and eosin (H&E) and Masson’s trichrome by conventional methods. Sections were imaged with a microscope coupled to a camera and viewed using Leica Aperio ImageScope software.

### Wheat Germ Agglutinin Staining

Hearts of the mice used for WGA staining were mounted on a Langendorff apparatus with gravity-driven flow (70–80 mmHg). The excised hearts were retrogradely perfused on the Langendorff apparatus with phosphate-buffered saline (PBS) until the perfusate flowed clear, and then the perfusion solution was switched to 10% neutral buffered formalin (NBF) containing 100 mmol/L KCl for approximately 10 min. After perfusion, the hearts were transferred to a conical tube containing approximately 20 ml of NBF and allowed to sit at room temperature for 1 h. The heart was then put into cassettes, cut transversely into four sections, and allowed to fix for 24 h in approximately 20 ml of fresh NBF. After 24 h, the hearts were transferred to 70% EtOH solution and allowed to sit at room temperature for 24 h and sent to the Veterinary Diagnostic Laboratory at the UIUC College of Veterinary Medicine for paraffin embedding and sectioning. To determine cell areas, the paraffin-embedded 3-μm-thick heart sections were taken, at 30-μm deep steps into the heart, for wheat germ agglutinin (WGA) staining. The sections were deparaffinized and rehydrated by placing the slides in 100% xylene twice for 3 min each, 1:1 xylene:EtOH once for 3 min, 100% EtOH twice for 3 min, 95% EtOH once for 3 min, 70% EtOH once for 3 min, 50% EtOH once for 3 min, and then placed in a bath of cool water. Antigen retrieval was performed by allowing the sections to sit in preheated antigen retrieval buffer (10 mmol/L sodium citrate, 0.05% tween-20, pH 6.0) at 95°C for 1 h. The slides were then removed from the antigen retrieval buffer, allowed to cool down and washed three times in PBS for 5 min. The slides were then incubated in FITC-conjugated WGA (Sigma L4895) at 10 μg/ml in PBS at room temp for 4 h. Next, the sections were washed three times for 5 min in PBS and mounted using ProLong Diamond antifade mountant (Invitrogen) and imaged the next day at the UIC Research Histology and Tissue Imaging core at the University of Illinois at Chicago using a Vectra multispectral microscope at 40 × magnification for quantification. A minimum of 10 different fields of view per biological replicate were used for the analysis. The cell areas were determined with Halo v2.3.2089.18 software at the Research Histology and Tissue Imaging core at the University of Illinois at Chicago.

### Hydroxyproline Assay

Hydroxyproline (HOP) content was determined as previously described (Flesch et al., 1997; Heydemann et al., 2005; Pena et al., 2010). Liquid nitrogen frozen mouse cardiac tissue was minced into a tared Pyrex 9826 screw cap vial to determine the wet weight of the tissue (15–22 mg). A standard curve of *trans-*4-hydroxy-L-proline (0–500 μM) was included in each assay to determine the μM HOP/mg of tissue.

### Assessment of Myofilament Phosphorylation by Pro-Q Diamond Stain

Mouse hearts were excised and immediately frozen in liquid nitrogen and stored at −80°C. Skinned myofibrils were prepared for phosphorylation analysis *via* Pro-Q Diamond and Coomassie staining as previously described with minor modifications (Layland et al., 2005). Briefly, 20 mg of left ventricular tissue was homogenized twice in 75 mM KCl, 10 mM imidazole pH 7.2, 2 mM MgCl_2_, 2 mM EGTA, 1% (v/v) Triton X-100 (Solaro et al., 1971), 1 mM NaN_3_ with protease and phosphatase inhibitors (Calbiochem #524624 1:100, Sigma #P-8340 1:100, and 500 nM Calyculin A) with a Dounce homogenizer. The homogenate was centrifuged at 16,000 × *g* for 1 min, and the pellets were washed once with the above buffer without Triton X-100 and centrifuged again. The pellets were solubilized in 8M urea, 2M thiourea, 0.05M Tris-HCl pH 6.8, 75 mM dithiothreitol (DTT) 3% sodium dodecyl sulfate (SDS), and 0.005% bromophenol blue (ISB buffer), and the protein concentration was determined with Pierce 660nm Protein Assay (Thermo Scientific) with the addition of the Ionic Detergent Compatibility Reagent (IDCR) following the manufacturer’s recommendations. Twelve percent sodium dodecyl sulfate-polyacrylamide gel electrophoresis (SDS-PAGE) gels (Fritz et al., 1989) were run to separate 5 μg of myofilament proteins per lane. The gels were stained with a phosphorylation-specific stain Pro-Q Diamond following the manufacturer’s recommendations (Invitrogen) and then subsequently stained with Coomassie G-250 following the manufacturer’s recommendations (BioRad). Gel images were captured with a Chemidoc MP (BioRad) and analyzed with Image Lab v6.0 and Microsoft Excel.

### Sodium Dodecyl Sulfate-Polyacrylamide Gel Electrophoresis for Myosin Heavy Chain Isoform Content

Myofilament protein samples prepared for the Pro-Q Diamond-stained gels were also used for the determination of myosin heavy chain isoform distribution. Myofilament protein (2.5 μg) was separated in 6% SDS-PAGE as previously described (Warren and Greaser, 2003). The gels were run in a Hoefer SE600 gel box and stained with Coomassie G-250 (BioRad) following manufacturer’s recommendations. Gel images were captured with a Chemidoc MP (BioRad) and analyzed with Image Lab v6.0 and Microsoft Excel.

### Immunoblot Analysis

Myofilament protein samples were also used for the determination of TnT-R92Q percent replacement. The TnT-R92Q has a myc-tag, which allows for a molecular weight separation versus the NTG-TnT isoforms 2 and 3. Myofilament protein (3 μg) was separated in 8% SDS-PAGE gels run in a Hoefer SE600 gel box at 22 mA with constant cooling (8°C) until the dye front was at the very bottom of the gel as previously described (Anderson et al., 1991). The gel was then trimmed to fit into a Criterion Transfer box (BioRad) and transferred onto 0.2 μm polyvinylidene fluoride (PVDF) membrane in 10 mM CAPS pH 11.0 (Matsudaira, 1987) buffer for 90 min at 20 V. The PDVF membrane was then probed with a TnT mouse monoclonal antibody 13-11 (Thermo Scientific #MS-295-P0) at 1:500 in 1% bovine serum albumin (BSA)-Tris-buffered saline with 0.1% Tween-20 (TBST).

Whole ventricular homogenates and skinned myofibrillar samples were prepared *via* an Omni International Bead Ruptor 24 Elite. The ventricular homogenates were ultimately solubilized in ISB buffer (see above), and the myofibrillar samples were prepared as previously (Hill et al., 2010; Patel et al., 2013; Utter et al., 2015) described for glutathione analysis and solubilized in 8M urea, 4% SDS, 0.05M Tris-HCl pH 6.8, and 0.005% bromophenol blue. A non-reducing 12% SDS-PAGE gel was run and transferred to 0.2 μm PVDF as above for the detection of glutathionylation (GSH) of MyBP-C and as previously described (Patel et al., 2013). To detect GSH, the membrane was first blocked in 5% non-fat dry milk-TBST with 2.5 mM NEM added. The membrane was then incubated with a mouse primary anti-GSH antibody (Virogen #101-A-250) 1:1,000 diluted in 5% non-fat dry milk-TBST. The membrane was then washed in TBST and incubated with a horseradish peroxidase (HRP)-conjugated mouse secondary antibody (Cell Signaling #7076S) 1:25,000 in 5% non-fat dry milk-TBST. The membrane was exposed with Clarity ECL reagent (BioRad).

All other Western blots were reducing 12 or 15% SDS-PAGE and transferred onto 0.2 μm PVDF membrane as described above. The membranes were incubated with various primary antibodies diluted in either 5% non-fat dry milk-TBST or 1% BSA-TBST: mouse sarco(endo)plasmic reticulum Ca^2+^ ATPase (SERCA)2a 1:1,000 (Abcam #2861), rabbit glyceraldehyde 3-phosphate dehydrogenase (GAPDH) 1:4,000 (Santa Cruz #sc-25778), rabbit phosphoSer16-phospholamban 1:1,000 (Millipore 07-052), mouse phospholamban clone A1 1:2,000 (Badrilla #A010-14), rabbit phosphoThr17-phospholamban 1:2,500 (Badrilla #A010-13AP), rabbit delta, beta, gamma-phosphoT287-CaMKII 1:1,000 (Invitrogen #PA5-37833), and rabbit delta CaMKII 1:5,000 (Badrilla #A010-56AP). After washing in TBST, the membranes were then detected with an HRP-conjugated mouse or rabbit secondary antibody (Cell Signaling #7076S or #7074S) 1:20,000–1:25,000 in 5% non-fat dry milk-TBST. Membranes were exposed to either Clarity ECL reagent (BioRad) or Pierce SuperSignal West Femto (Thermo Scientific #34094) and imaged with a Chemidoc MP (BioRad) and analyzed with Image Lab v6.0 and Microsoft Excel.

### Statistical Analysis

Gaussian distribution was assessed using the Shapiro–Wilk test and equal variance using Brown–Forsythe test. Data that show normal distribution and equal variance were analyzed using a one-way ANOVA followed by Tukey’s *post hoc* test for multiple comparisons. Data that show normal distribution and unequal variance were analyzed using Brown–Forsythe and Welch ANOVA tests followed by Tamahane T2 test or unpaired *t*-test with Welch’s correction for multiple comparisons. Otherwise, non-parametric Kruskal–Wallis test followed by Dunn’s test for multiple comparisons were used. When two groups were compared, unpaired *t*-test was used. All data are presented as mean ± SE. Significance was set at *P* < 0.05 and marked as ^∗^ for *P* < 0.05, ^∗∗^ for *P* < 0.01, and ^∗∗∗^ for *P* < 0.001. All statistical analyses were performed using GraphPad Prism 8.0 Software (GraphPad, Inc., La Jolla, CA, United States).

## Results

### Baseline Cardiac Function and Morphology of Transgenic/Phospholamban Mice in FVB/N Background

**FIGURE 1:**
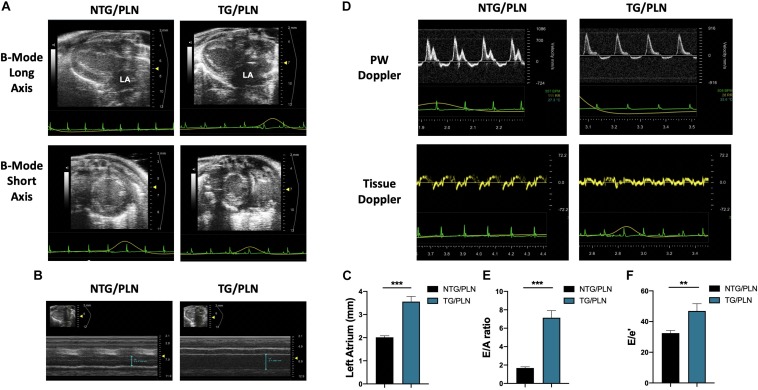
Echocardiographic measurements show atrial enlargement and diastolic dysfunction in transgenic (TG)/phospholamban (PLN) mice. **(A)** Representative B mode images of parasternal long axis (top) and short axis (bottom). **(B)** Long axis M-mode images of aorta and left atrium (LA). **(C)** Summary data of LA size in non-transgenic (NTG)/PLN and TG/PLN mice. **(D)** Pulsed-wave Doppler of mitral flow (top) and tissue Doppler images (bottom) of the mitral annulus obtained from echocardiography. Summary data of peak velocity blood flow in early diastole (the E wave) to peak velocity flow in late diastole (A wave) (E/A) ratio **(E)** and peak velocity blood flow in early diastole (E wave) to peak mitral annular velocity during early filling (e′) (E/e′) ratio **(F)** in NTG/PLN and TG/PLN mice. Summary data are presented as means ± SEM. Differences among groups were analyzed by Mann–Whitney test. *N* = 13 (NTG/PLN) and 11 (TG/PLN) per group. ^∗^*P* < 0.05, **P < 0.01, ***P < 0.001.

To determine the influence of the genetic background on the HCM-linked phenotype, we crossed TnT-R92Q C57/Bl mice (Tardiff et al., 1999) into the FVB/N background for 10 generations as described in Section “Materials and Methods.” Baseline cardiac function and morphology of the TG/PLN mouse model in the FVB/N background was examined using high-resolution echocardiography. Echocardiography revealed that at 16 weeks of age, mice display severe atrial remodeling and diastolic dysfunction, but no left ventricular hypertrophy (Figure 1 and Table 1). Figure 1 shows representative B-mode images of parasternal long (panel A, top) and short axis (panel A, bottom), M-mode images in long axis at the level of the aortic root and atria (panel B), and average LA size (panel C) in NTG/PLN and TG/PLN mice. The representative images of pulsed-wave Doppler of mitral flow and tissue Doppler images of mitral annulus in NTG/PLN and TG/PLN mice are shown in panel D. The LA size was significantly enlarged in TG/PLN mice compared to NTG/PLN mice (Figure 1C), but no changes were found in left ventricular internal diastolic dimension (LVIDd), LV mass, or relative wall thickness (RWT) between the NTG/PLN and TG/PLN groups (Table 1). However, TG/PLN hearts showed significant diastolic dysfunction as assessed by an increase in E/A ratio (the ratio of early to late diastolic mitral flow), an increase in the E/e′ ratio (ratio of early diastolic mitral flow to early diastolic mitral annulus velocity), and e′ (Figures 1E,F and Table 1). The global systolic function was preserved as no significant changes were observed between the ejection fraction (EF) and the velocity of circumferential shortening (V_cf_). However, we found depressed peak systolic annular velocity (S′) in the TG/PLN mice compared to the NTG/PLN mice (Table 1).

**TABLE 1:** Echocardiographic assessment of cardiac function.

**Parameter/group**	**NTG/PLN**	**TG/PLN**	**NTG/PLNKO**	**TG/PLNKO**
Sample size (n)	13	12	13	14
LA (mm)	2.010.06	3.560.22***	1.900.10^‡⁣‡‡^	2.060.05^‡⁣‡‡^
LVIDd (mm)	3.760.10	3.840.15	3.820.07	3.800.12
LV mass (mg)	74.522.99	89.085.08	84.204.92	75.723.39
RWT	0.390.03	0.430.03	0.400.01	0.400.03
E/A ratio	1.680.12	7.130.78***	1.760.10^‡⁣‡‡^	1.950.10^‡‡^
E/e′ ratio	−32.481.68	−46.924.71*	−30.111.10^‡‡^	−32.301.38^‡^
E (mm/s)	797.737.61	716.136.79	842.036.10	836.630.00
e′ (mm/s)	22.971.01	15.561.58*	27.081.02^‡⁣‡‡^	26.291.16^‡⁣‡‡^
IVRT (ms)	13.090.88	14.210.85	10.940.46^‡^	12.570.43
DT (ms)	21.700.88	23.201.47	21.801.08	20.600.69
HR (beats/min)	437.214.63	445.826.01	484.212.87	458.215.90
SV (μl)	41.331.63	45.693.50	40.751.72	45.783.04
EF (%)	67.512.08	67.952.23	63.851.58	70.071.70
S′ (mm/s)	21.411.11	13.991.53***	24.500.88^‡⁣‡‡^	24.170.94^‡⁣‡‡^
V_cf_ (circ/s)	6.770.30	6.430.28	7.650.27^‡^	7.480.33

Non-transgenic (NTG)/phospholamban (PLN), mice express wild-type troponin T (TnT) and normal level of PLN; NTG/phospholamban knockout (PLNKO), mice express wild-type TnT and no PLN; transgenic (TG)/PLN, mice express TnT-R92Q and normal level of PLN; TG/PLNKO, mice express TnT-R92Q and no PLN. LA, left atrial dimension; LV mass, left ventricular mass; LVIDd, left ventricular internal diastolic dimension; RWT, relative wall thickness; E/A, peak velocity blood flow in early diastole (the E wave) to peak velocity flow in late diastole (A wave); E/e′, peak velocity blood flow in early diastole (E wave) to peak mitral annular velocity during early filling (e′); IVRT, isovolumetric relaxation time; DT, E wave deceleration time; HR, heart rate; SV, stoke volume; EF, ejection fraction; S′, peak systolic annular velocity; V_cf_, velocity of circumferential shortening. Data are presented as mean ± SEM (n, number of animals). Differences between groups in LVIDd, LV mass, E, EF, V_cf_, S′, and DT were analyzed by one-way ANOVA followed by Tukey’s multiple comparison test. Differences between groups in HR, LA, RWT, E/A, E/e′, e′, and IVRT were analyzed using non-parametric Kruskal–Wallis test followed by Dunn’s test for multiple comparisons. For SV, data were analyzed by Brown–Forsythe and Welch ANOVA test followed by Dunnett’s multiple comparison test. *Significantly different vs. NTG/PLN, ^‡^significantly different vs. TG/PLN. *** or ^‡‡‡^*P* < 0.001, ** or ^‡‡^*P* < 0.01, * or ^‡^*P* < 0.05.

### Cardiac Function and Morphology Are Normal in Transgenic/Phospholamban Knockout Mice

**FIGURE 2:**
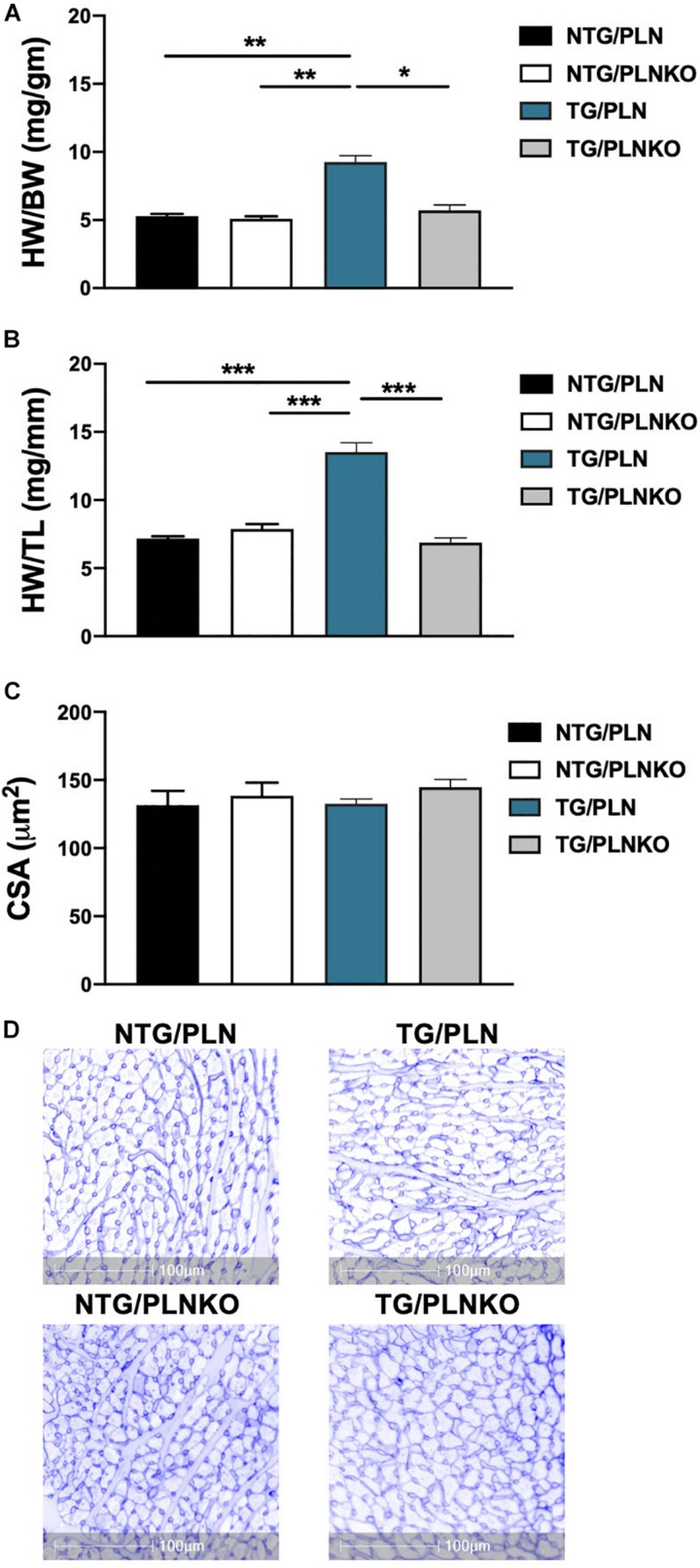
Evaluation of cardiac hypertrophy. Summary data of **(A)** heart weight to body weight (HW/BW) ratio, **(B)** heart weight to tibia length (HW/TL) ratio, and **(C)** cross-sectional area (CSA) of cardiac myocytes calculated from cardiac cross section stained with wheat germ agglutinin (WGA). **(D)** Representative images of cardiac cross section stained with WGA. Data are presented as means ± SEM. Differences among HW/TL and CSA groups by one-way ANOVA followed by Tukey’s multiple comparison test. Differences among HW/BW groups were determined using Kruskal–Wallis test followed by Dunn’s multiple comparison test. *N* = 6–11 per group. *P < 0.05, **P < 0.01, ^∗^**P < 0.001.

To test the hypothesis that increased SR Ca^2+^ uptake can prevent the HCM phenotype in TG TnT-R92Q mice, we crossed the TnT-R92 with PLNKO mice to generate mutant mice that do not express PLN (TG/PLNKO). Echocardiography showed that at 16 weeks of age, TG/PLNKO mice show normal morphology (LA size, LV mass, LVIDd, and RWT) and diastolic (E/A ratio, E/e′, IVRT, and DT) and systolic function (EF, S′, and V_cf_) (Table 1). TG/PLNKO mice also showed no changes in the HW/BW and HW/TL ratios compared to the NTG/PLN and NTG/PLNKO groups (Figures 2A,B). In addition to our finding of no changes in LV mass among all four groups (Table 1), the sizes (cross-section areas) of ventricle cardiac myocytes were not different between all four groups (Figures 2C,D).

**FIGURE 3:**
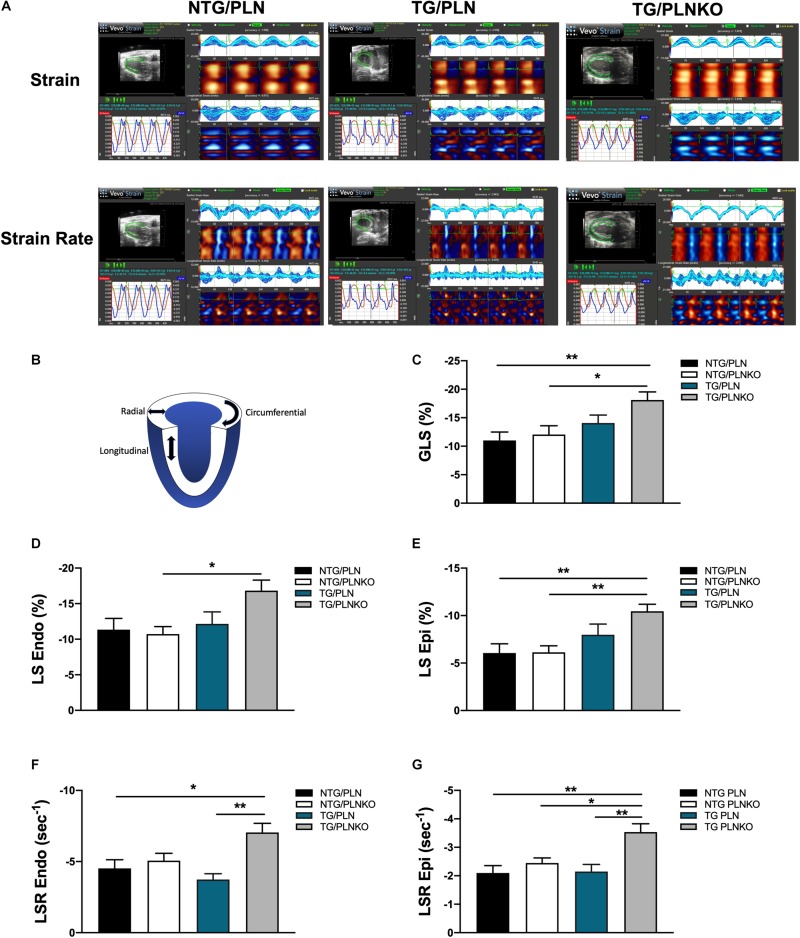
Longitudinal strain (LS) measurements by echocardiography. **(A)** Representative images of speckle-tracking echocardiography in non-transgenic (NTG)/phospholamban (PLN), transgenic (TG)/PLN, and TG/phospholamban knockout (PLNKO) mice. **(B)** Schematic drawing of left ventricle with indication of longitudinal (LS), radial, and circumferential strain measurements. Summary of **(C)** global LS (GLS), **(D)** LS in endocardium (LS Endo), **(E)** LS in epicardium (LS Epi), **(F)** LS rate (LSR) in endocardium (LSR Endo), and **(G)** LSR in epicardium (LSR Epi) in the NTG/PLN, NTG/PLNKO, TG/PLN, and TG/PLNKO groups. Data are presented as means ± SEM. *N* = 7–12. Differences among groups were analyzed by one-way ANOVA followed by Tukey’s multiple comparison test. *P < 0.05, **P < 0.01, ***P < 0.001.

**FIGURE 4:**
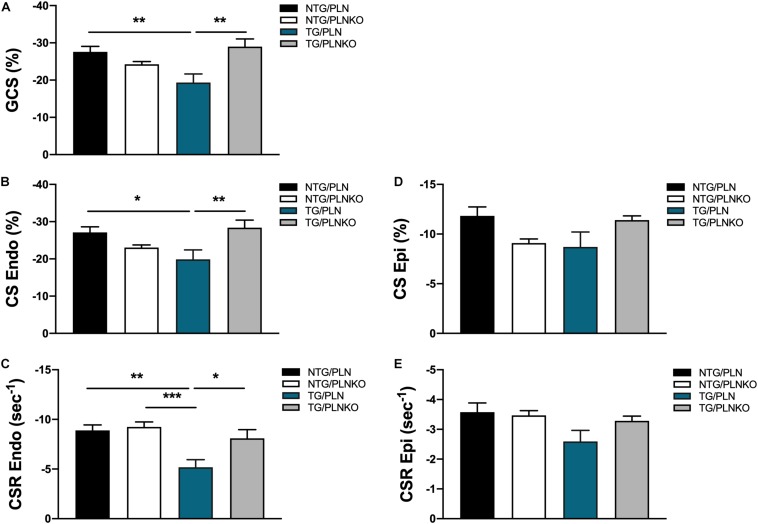
Circumferential strain (CS) measurements by echocardiography. Summary of **(A)** global CS (GCS), **(B)** CS in endocardium (CS Endo), **(C)** CS rate in endocardium (CSR Endo), **(D)** CS in epicardium (CS Epi) and **(E)** CSR in epicardium (CSR Epi) in non-transgenic (NTG)/phospholamban (PLN), NTG/phospholamban knockout (PLNKO), transgenic (TG)/PLN and TG/PLNKO groups. Data are presented as means ± SEM. Differences among GCS, CS Endo, CSR Endo and CSR Epi groups were analyzed by one-way ANOVA followed by Tukey’s multiple comparisons test. Differences among CS Ep groups were analyzed using Kruskal-Wallis test followed by Dunn’s multiple comparison test. *N* = 7–12 *p < 0.05, **P < 0.01, ***P < 0.001.

**FIGURE 5:**
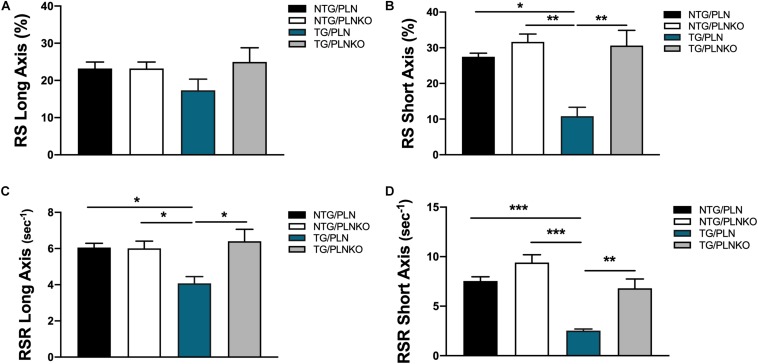
Radial strain measurements by echocardiography. Summary of **(A)** radial strain (RS) in long axis, **(B)** RS in short axis, **(C)** radial strain rate (RSR) in long axis, and **(D)** radial strain rate (RSR) in short axis. Data are presented as means ± SEM. Differences among RS, RS in short axis, and RSR in long axis groups were analyzed by one-way ANOVA followed by Tukey’s multiple comparison test. Differences among RSR in short axis were analyzed using Brown–Forsythe and Welch ANOVA tests followed by Dunnett’s T3 test for multiple comparisons. *N* = 7–12. *P < 0.05, **P < 0.01, ***P < 0.001.

In order to further investigate potential regional changes in systolic function among the mouse groups, we performed speckle tracking-based strain analysis using the Vevo2100 system. Figure 3A shows original recordings of strain and strain rate obtained in NTG/PLN, TG/PLN, and TG/PLNKO mice. We calculated longitudinal (LS), radial (RS), and circumferential strain (CS) and strain rate (LSR, RSR, and CSR, respectively) (Figure 3B). Analysis did not reveal significant changes between the TG/PLN and NTG/PLN groups in global LS (GLS), LS in endocardium, LS in epicardium, LS rate in endocardium (LSR), or LSR in epicardium (Figures 3C–G). However, TG/PLNKO hearts showed increased LSR in the endocardium and epicardium when compared to TG/PLN hearts (Figures 3F,G). Although LS measurements (Figure 3) did not show any changes between the NTG/PLN and TG/PLN groups, CS measurements (Figure 4) revealed reduced global CS (GCS) (panel A), CS in endocardium (CS Endo, panel B), and CSR in the endocardium (CSR Endo, panel C) in the TG/PLN group, but no changes in CS and CSR in the epicardium (panels D, E). RS in the long axis was not significantly reduced in the TG/PLN group compared to the NTG/PLN group (Figure 5A). Nonetheless, RS in the short axis (Figure 5B) and RSR in both the long and short axes (Figures 5C,D) were depressed in the TG/PLN group. Importantly, PLN ablation was able to restore all the parameters to normal levels in TG mice (Figures 5B–D).

### Fibrosis Is Upregulated in Troponin T-R92Q Mice but Not Altered in Transgenic-Phospholamban Knockout Mice

**FIGURE 6:**
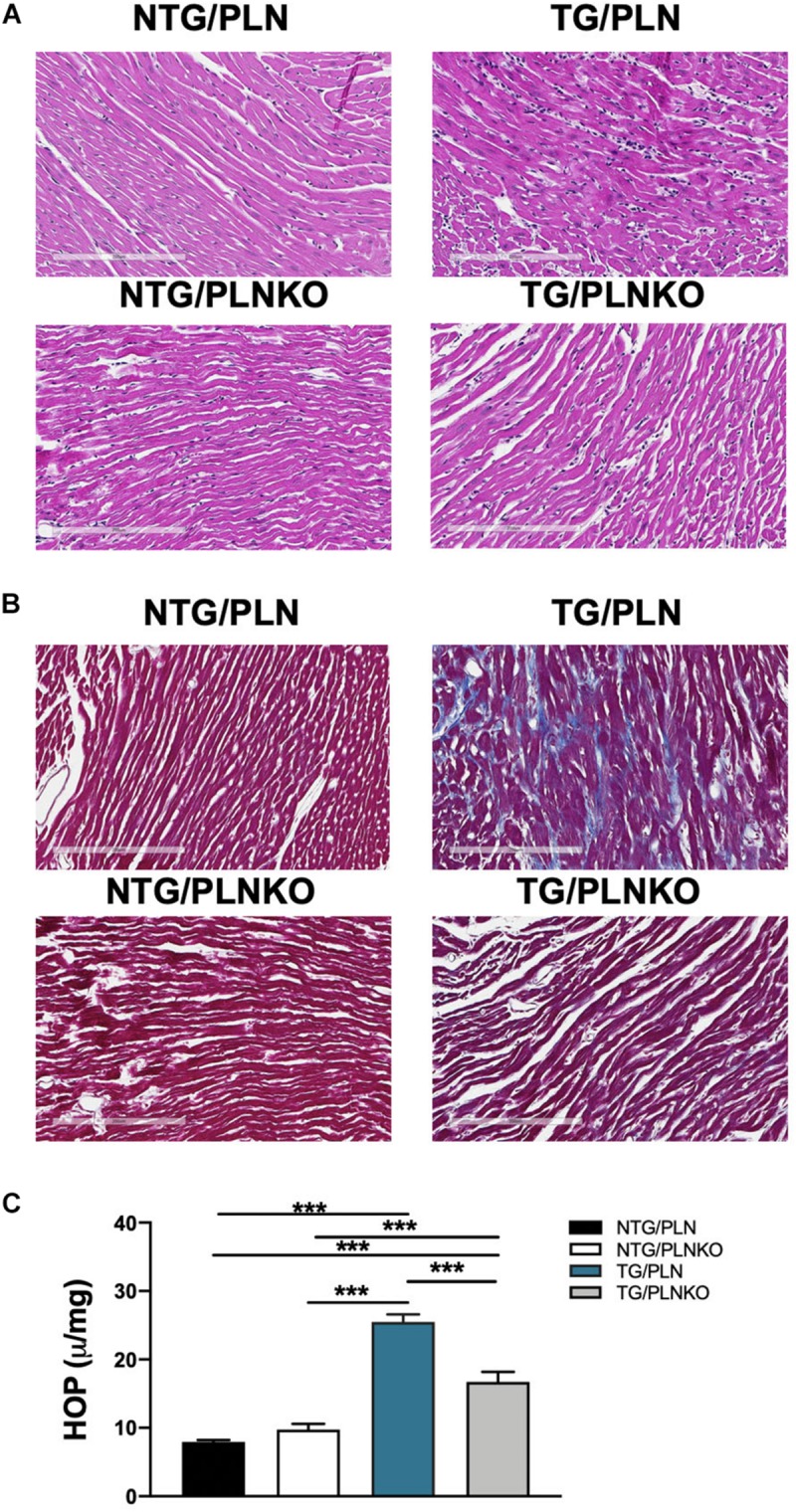
Histology and hydroxyproline (HOP) assay of non-transgenic (NTG)/phospholamban (PLN), NTG/phospholamban knockout (PLNKO), transgenic (TG)/PLN, and TG/PLNKO mouse hearts. Representative left ventricular (LV) heart sections stained with **(A)** hematoxylin and eosin and **(B)** Masson’s trichrome (MT). The intense myocyte disarray is depicted in H&E-stained sections, and extensive fibrosis is observed in MT-stained sections (blue stain) in TG/PLN hearts. TG/PLNKO hearts show less disarray and fibrosis than TG/PLN hearts. **(C)** Hydroxyproline content. Data are presented as means ± SEM. *N* = 6 per group. Differences among groups were analyzed by one-way ANOVA followed by Tukey’s multiple comparison test. *P < 0.05, **P < 0.01, ***P < 0.001.

Since both atrial morphology and diastolic function were altered in TG/PLN mice but remained normal in TG/PLNKO mice, we tested whether there is a difference in the degree of fibrosis between the groups. Histological analysis showed myocyte disarray and increased collagen deposition in TG/PLN hearts compared to other groups (Figures 6A,B). Increased collagen content seen in histological sections in TG/PLN mice quantified with the HOP assay showed increased HOP content only in TG/PLN hearts (Figure 6C). Taken together, these data indicate an increase in fibrosis only in TG/PLN mice.

### Phospholamban Knockout Does Not Alter Myofilament Response to Ca^2+^

**FIGURE 7:**
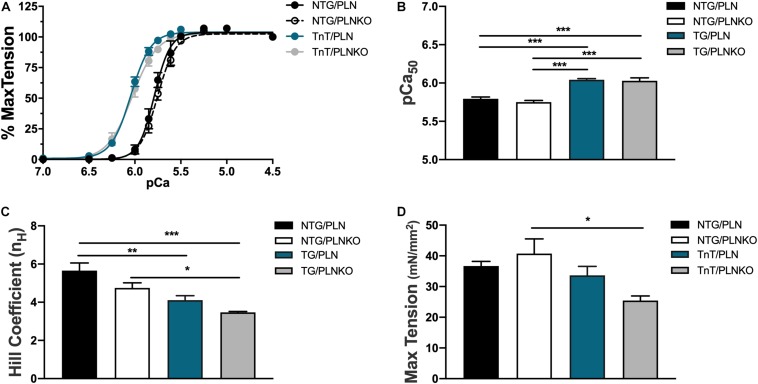
The myofilament Ca^2+^ response is increased in transgenic (TG) mice. **(A)** Force–Ca^2+^ relationship of fibers isolated from non-transgenic (NTG)/phospholamban (PLN), NTG/phospholamban knockout (PLNKO), TG/PLN, and TG/PLNKO mice. Summary data of **(B)** myofilament Ca^2+^ sensitivity [Ca^2+^ concentration of half-maximal activation (pCa_50_)], **(C)** cooperativity measured by the Hill coefficient (n_H_), and **(D)** maximal tension generation. Data are presented as means ± SEM. *N* = 5–6. Differences among groups were analyzed by one-way ANOVA followed by Tukey’s multiple comparison test. *P < 0.05, **P < 0.01, ***P < 0.001.

To test whether altered myofilament sensitivity contributes to the functional and morphological improvement in TG/PLNKO mice, we measured the force–Ca^2+^ relationship in detergent-extracted fiber bundles from papillary muscles in NTG/PLN, NTG/PLNKO, TG/PLN, and TG/PLNKO mice (Figure 7). Consistent with previous reports on the TnT-R92Q mouse model in the C57BL/6 genetic background (Chandra et al., 2001), myofilaments from TG/PLN hearts showed a significantly increased Ca^2+^ sensitivity compared to myofilaments from NTG/PLN mice [pCa_50_ = 6.04 ± 0.02 (*n* = 5) vs. 5.79 ± 0.02 (*n* = 5)]. PLNKO did not result in altered myofilament Ca^2+^ sensitivity in either group (pCa_50_ = 5.75 ± 0.02, *n* = 6 in NTG/PLNKO mice and 6.03 ± 0.04, *n* = 5 in TG/PLNKO mice). Increased myofilament Ca^2+^ sensitivity observed in the TG groups was associated with a reduction in cooperativity, as measured by the Hill coefficient (Figure 7C) and decreased max tension only in the TG/PLNKO group (Figure 7D).

### Phosphorylation of Cardiac Myofilament Proteins Is Not Altered, but Expression of β-MHC Isoform Is Increased in Transgenic/Phospholamban Mice

**FIGURE 8:**
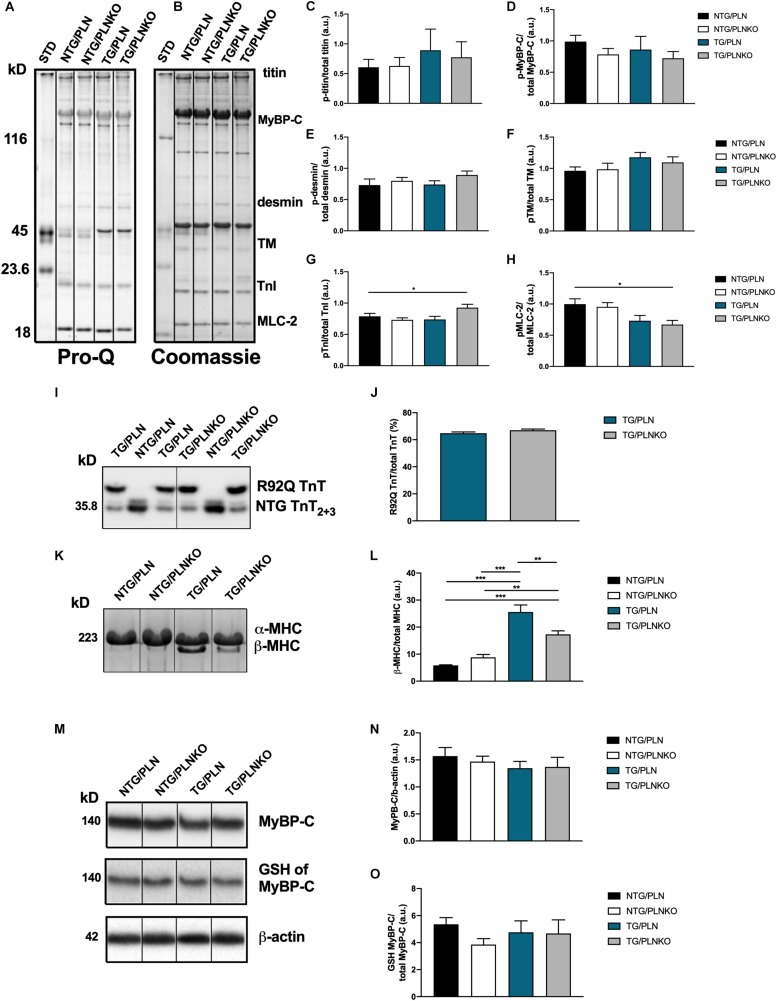
Myofilament isoform abundance and modifications in non-transgenic (NTG)/phospholamban (PLN), NTG/phospholamban knockout (PLNKO), transgenic (TG)/PLN, and TG/PLNKO mice. **(A)** Representative Pro-Q gel image. **(B)** Representative Coomassie blue gel image. Summary of phosphorylation data of **(C)** titin, **(D)** myosin binding protein C (p-MyBP-C), **(E)** desmin, **(F)** tropomyosin (TM), **(G)** troponin I (TnI), and **(H)** myosin light chain 2 (MLC-2). Phosphorylation of each myofilament protein was calculated from Pro-Q image **(A)** and normalized to total abundance of these proteins from Coomassie gel **(B)**. **(I,J)** Abundance of mutated TnT-R92Q protein in TG/PLN and TG/PLNKO hearts. TnT_2__+__3_ refers to isoforms of TnT, and the R92Q TnT has a myc-tag that allows for a molecular weight distinction with the NTG TnT. **(K)** Sodium dodecyl sulfate–polyacrylamide gel electrophoresis (SDS-PAGE) Coomassie blue gel image of myosin heavy chain (MHC) isoforms. **(L)** Summary data of MHC isoforms. **(M)** Western blot analysis of myosin binding protein C (MyBP-C), S-glutathionylation (GSH) of MyBP-C and β-actin. **(N)** Summary abundance of MyBP-C. **(O)** Summary data of GSH of MyBP-C. Data are presented as means ± SEM. *N* = 4–6. Differences among four groups were analyzed by one-way ANOVA followed by Tukey’s multiple comparison test. If only two groups were compared, unpaired *t*-test was used. *P < 0.05, **P < 0.01, ***P < 0.001.

The expression of different myofilament isoforms and their post-translational modifications are known to affect myofilament properties (Kobayashi and Solaro, 2005; van der Velden and Stienen, 2019) and could contribute to the recovered cardiac function. We therefore investigated the phosphorylation status of myofilament proteins (Figure 8A). There were no changes in titin, tropomyosin, MyBP-C, or desmin phosphorylation among all groups. However, there was an increase in TnT phosphorylation and a decrease in phosphorylation of MLC2 in the TG/PLNKO group compared to the NTG/PLN group (Figures 8B–H), indicating the presence of some compensatory mechanisms. Since the expression of TnT-R92Q may alter myofilament Ca^2+^ sensitivity, we assessed the levels of abundance of TnT-R92Q in TG/PLN and TG/PLNKO hearts but found that both groups had similar levels of TnT-R92Q (Figures 8I,J). In previous studies with another model of HCM (Tm-E180G) (Ryba et al., 2019), we showed an increased expression of the fetal β-MHC isoform. We, therefore, tested MHC isoform population in all four groups of mice. We found a significant expression of the fetal β-MHC isoform in TnT/PLN mice, which was reduced in the TnT/PLNKO group, but did not reach levels seen in NTG groups (Figures 8K,L). We have also previously reported that S-glutathionylation of cMyBP-C is increased in Tm-E180G mice and contributes to the increased myofilament Ca^2+^ sensitivity. Thus, we tested the level of S-glutathionylation of cMyBP-C in the NTG and TG groups but found no changes between groups (Figures 8M–O).

### Expression of Sarco(endo)plasmic Reticulum Ca^2+^ ATPase (SERCA)2a, Phospholamban, and Ca^2+^/Calmodulin-Dependent Protein Kinase II

**FIGURE 9:**
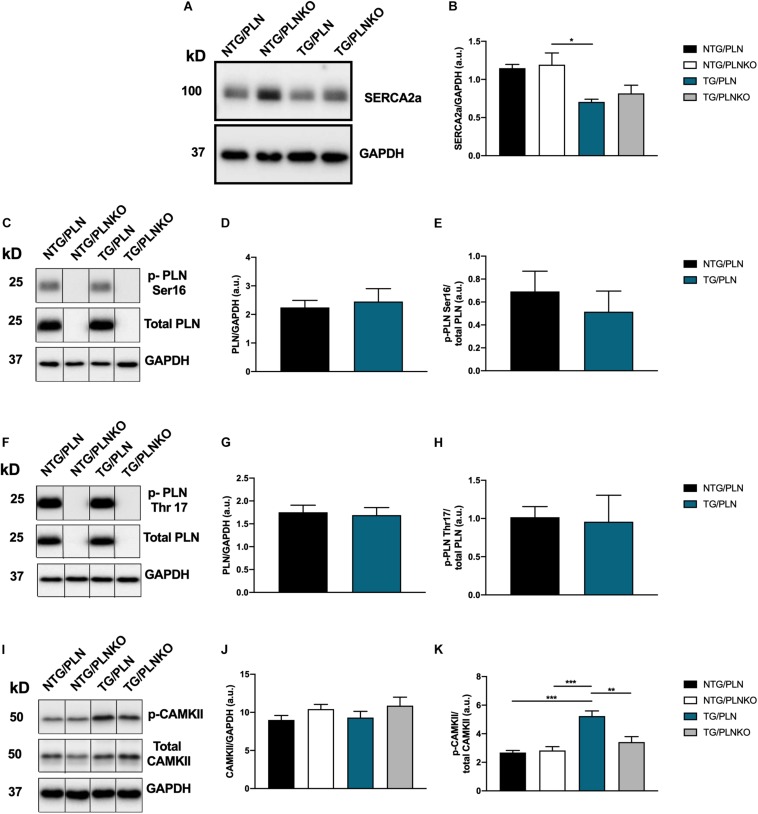
Expression of Ca^2+^ regulatory proteins. **(A)** Representative Western blot of sarco(endo)plasmic reticulum Ca^2+^ ATPase (SERCA)2 and glyceraldehyde 3-phosphate dehydrogenase (GAPDH), and **(B)** summary data of SERCA2a show no significant changes between transgenic (TG)/phospholamban (PLN) and TG/phospholamban knockout (PLNKO) groups. **(C)** Representative Western blot of phosphorylation PLN expression and phosphorylation at Ser16 (p-PLN Ser16) and GAPDH. Summary data show no significant changes in PLN abundance **(D)** and p-PLN Ser16 **(E)** between the non-transgenic (NTG)/PLN and TG/PLN groups. **(F)** Representative Western blot of PLN expression and phosphorylation at Thr17 (p-PLN Thr17) and GAPDH. Summary data show no significant changes in PLN expression **(G)** and p-PLN Thr17 **(H)** between the NTG/PLN and TG/PLN groups. **(I)** Representative Western blot of CaMKII abundance, phosphorylation (T287), and GAPDH. Summary data show no significant changes in CaMKII expression **(J)** but increased phosphorylation of CaMKII **(K)** in TG/PLN group. Data are presented as means ± SEM. *N* = 4–6. Differences among groups were analyzed by one-way ANOVA followed by Tukey’s multiple comparison test. If only two groups were compared, unpaired *t*-test was used. *P < 0.05, **P < 0.01, ***P < 0.001.

Modification or expression of the Ca^2+^-regulatory proteins PLN and SERCA has been implicated in the regulation of inotropy and lusitropy in the heart. We investigated the level of SERCA expression in NTG/PLN, NTG/PLNKO, TG/PLN, and TG/PLNKO hearts (Figures 9A,B) and found a significant reduction in SERCA2 expression in the TG/PLN group only compared to the NTG/PLNKO group. No differences in the expression of PLN or phosphorylation at Ser16 and Thr17 were found between the NTG/PLN and TG/PLN groups (Figures 9C–H).

Changes in CaMKII signaling have been previously reported in human and mouse models of HCM (Helms et al., 2016; Lehman et al., 2019). We, therefore, also assessed the levels of expression and phosphorylation of CaMKII in our mice. The total abundance of CaMKII was not different among these groups. However, phosphorylation of CaMKII was significantly increased in the TG/PLN group compared to the other NTG groups but was normalized by PLN ablation in the TG/PLNKO group (Figures 9I–K).

## Discussion

A major novel finding from experiments reported here is the demonstration that PLNKO was able to prevent the development of the HCM disorder in the TG mouse model without altering the increases in myofilament Ca^2+^ sensitivity or their phosphorylation. Our results strongly suggest that the rescue of the HCM phenotype involved an increased activity of SERCA2 that resulted in normal relaxation, prevention of an increased phosphorylation of CaMKII, development of fibrosis, and diminished activation of the “fetal/hypertrophic gene program.” We also found that compared to standard systolic echo parameters such as EF, speckle strain measurements provide a more sensitive approach to detect systolic dysfunction in our model of HCM.

An important aspect of our findings is that mice expressing mutated TnT-R92Q in FVB/N genetic background develop profound atrial dilation without LV hypertrophy compared to smaller LV size (Tardiff et al., 1999) or septal hypertrophy (Coppini et al., 2017) previously reported in C57BL/6 genetic background most likely due to differential presence of modifying genes in these two genetic backgrounds. Moreover, more severe diastolic dysfunction and systolic dysfunction, observed by speckle-tracking echocardiography, were observed compared to the originally reported about 20% decrease in diastolic function and 18% improvement in systolic function measured in working heart preparation (Tardiff et al., 1999). Recently, Ferrantini et al. (2017) using echocardiography reported a small (about 5%) increase in EF and reduced E/A ratio from about 1.4 to 1.0 in mice with TnT-R92Q mutation. Interestingly, independent of the genetic background, hearts with the TnT-R92Q mutation show increased fibrosis (Tardiff et al., 1999; Ferrantini et al., 2017) and myofilament Ca^2+^ sensitivity (delta pCa_50_ = 0.28) (Chandra et al., 2001), which are all characteristics of HCM in both human and mouse models (Ashrafian et al., 2011; Frey et al., 2012). Our findings demonstrate that the genetic background of the mice influences the HCM phenotype caused by TnT-R92Q mutation. Moreover, it has been previously presented in two HCM mouse models that mice expressing tropomyosin (Tm-E180G) developed a more pronounced hypertrophic phenotype in FVB/N than in C57BL/6 genetic background (Prabhakar et al., 2001; Michele et al., 2002), and in mice with a mutation in actin (ACTZ E99K), the probability of sudden cardiac death was almost completely eliminated in mice bred on C57/BL6 background (Rowlands et al., 2017). The importance of the mouse genetic background on the basal cardiac function, adaptations to exercise, or stress is also well-documented (Barnabei et al., 2010; Peng et al., 2011; Gibb et al., 2016).

Increased myofilament Ca^2+^ sensitivity observed in HCM can be directly targeted or counterbalanced by alteration of Ca^2+^ fluxes (Alves et al., 2010). We (Alves et al., 2014; Warren et al., 2015) and others (Tadano et al., 2010) have previously reported the beneficial effects of targeting directly the myofilament Ca^2+^ sensitivity in HCM. Since on the cellular level, diastolic function is regulated by both myofilament Ca^2+^ responsiveness and Ca^2+^ fluxes, several studies targeted mechanisms involved in E-C coupling. It has been shown that L-type Ca^2+^ channel blocker, diltiazem, has a favorable effect in HCM with mutation in alpha MHC (Arg403Gln) (Semsarian et al., 2002), and buffering of Ca^2+^ with parvalbumin corrected slower relaxation in adult cardiac myocytes expressing mutated Tm (Tm-E180G, Tm-A63V) (Coutu et al., 2004). We have previously shown that HCM Tm-E180G mice can be rescued either by partially restoring myofilament Ca^2+^ sensitivity (Alves et al., 2014) or by increased SERCA2 activity by PLNKO or increased expression of SERCA2 (Pena et al., 2010; Gaffin et al., 2011). Data from these experiments indicated that SERCA2 activity may be a good target for HCM linked to mutations associated with increased myofilament Ca^2+^ sensitivity, but PLNKO failed to rescue hypertrophy caused by a truncation in thick filament, MYBP-C (Song et al., 2003). Moreover, the potential benefits of PLN ablation have not been directly tested in any other model with mutations in thin filament proteins besides the Tm-E180G HCM mouse. TG TnT-R92Q C57BL/6 mice were reported to show decreased SERCA2 and increased CaMKII activity that resulted in an increased level of diastolic Ca^2+^ (Ferrantini et al., 2017). In our current studies, differences in the expression levels of SERCA2 between the NTG/PLN and TG/PLN groups did not reach significance, although we saw a clear trend toward lower levels in the TG/PLN group with a significant difference between the TG/PLN and NTG/PLNKO groups. These results indicate potential dysregulation of E-C coupling and successful rescue of the TnT-R92Q model of HCM in the FVB/N background.

In addition, consistent with the previous report in mice expressing the same mutation (Ferrantini et al., 2017), we found a significantly increased phosphorylation level of CaMKII, which was completely normalized in mice rescued by PLNKO (TG/PLNKO). Chronic activation of CaMKII was recently reported in human samples from mutation-positive HCM (Helms et al., 2016) and in other HCM mouse models with TnT-R92L and TnT-R92W mutations (Lehman et al., 2019). These data suggest that CaMKII activation plays a role in progression of the disease in HCM and may serve as a therapeutic target, but only for a particular cohort of patients with specific mutations, since inhibition of CaMKII resulted in recovery of diastolic function only in the R92W but not in R92L mice (Lehman et al., 2019). Why inhibition of CaMKII only works for some mutations is not completely understood. Another intriguing finding is that in TG/PLN mice, activation of CaMKII did not result in altered phosphorylation of PLN at Thr17, that was previously reported in ketoconazole-treated TnT-R92Q C57BL/6 mice (Coppini et al., 2017) and in human samples from mutation-positive HCM patients (Helms et al., 2016). Phosphorylation level of CaMKII was normal in hearts of mice rescued by PLNKO. We think that the most likely mechanism for the normalization is an increased SR Ca^2+^ uptake and improved relaxation that led to a reduction in local mechanical strain caused by R92Q mutation. Along these lines, it has been proposed that increased mechanical strain observed in TnT-R92Q mice results in activation of CaMKII (Jian et al., 2014). It is also possible that initially, there is an increase in PLN Thr17 associated with improved systolic function, but during progression of the disease, the level of PLN phosphorylation decreases as the heart remodels and systolic function starts to decline as we observed in this study.

Our data support the hypothesis that speckle-tracking strain measurements especially circumferential and radial parameters, which indicate impairment in systolic function, may be a more sensitive early marker for systolic dysfunction in HCM. We show that at 16 weeks of age, systolic function based on EF is not altered in TG/PLN mice compared to other groups, whereas there is a significant change in strain parameters. Use of the speckle-tracking technique to detect mechanical dyssynchrony before organ failure in a K_ATP_ channel-knockout mouse model of dilated cardiomyopathy has been previously reported by Yamada et al. (2013). The authors showed evidence that speckle-tracking echocardiography was able to detect myocardial changes at a time point when standard echocardiographic parameters did not detect any changes. RS, LS, and reverse LS compared to conventional echocardiographic measurements were also reported to be more sensitive to detect early changes in LV diastolic and systolic function in aging (de Lucia et al., 2019). On the other hand, Peng et al. (2009) have previously reported that TAC-induced heart failure and fibrotic changes were better tracked by CS measurements rather than RS measurements. These conflicting data may suggest that a full range of strain-derived measurements should be included in addition to conventional echo parameters in early detection of cardiac dysfunction. Moreover, recent human data showed that strain measurements permit detection of abnormalities in myocardial mechanics before the development of clinical hypertrophy (Williams et al., 2018). Data also indicate that abnormal GLS is an independent factor associated with poor cardiac outcomes (Reant et al., 2016; Liu et al., 2017).

Improvement in diastolic function in TG/PLNKO mice was not associated with significant changes in myofilament Ca^2+^ sensitivity. This lack of change in myofilament Ca^2+^ sensitivity correlates well with the lack of changes in myofilament phosphorylation and in no increase in S-glutathionylation of MyBP-C. Interestingly, in contrast to our findings with the TnT-R92Q mice, the Tm-E180G HCM mouse model demonstrated increased oxidative stress and expression of NOX2 that resulted in S-glutathionylation of MyBP-C. The result was a further increase in myofilament Ca^2+^ sensitivity that was rescued by treatment with FTY720 (fingolimod) (Ryba et al., 2019) or NAC treatment (Wilder et al., 2015). FTY720 treatment improved diastolic function mainly through reversal of S-glutathionylation of MyBP-C without affecting fibrosis. Here, we did not find changes in myofilament Ca^2+^ sensitivity, but prevention of fibrosis. These data suggest that several mechanisms contribute to diastolic dysfunction in HCM, and their contribution and therefore treatment may be mutation dependent.

In summary, our results indicate that targeting diastolic dysfunction through altering Ca^2+^ fluxes was able to prevent the development of the HCM phenotype and should be considered as a potential new therapeutic target for HCM patients. Current therapies involve a focus on sarcomere myosin de-activators such as mavacamten, developed largely based on mutations and truncations in the thick filament proteins (Green et al., 2016). However, as emphasized by Maron et al. (2019), the heterogeneity of the pathobiology and genetics in HCM may require approaches in precision medicine. These approaches permit formulation of a network medicine analysis with aspects of personalized medicine in which it is likely that therapies may include both sarcomere tension-directed therapies and therapies altering Ca^2+^ fluxes.

## Data Availability Statement

The datasets generated for this study are available on request to the corresponding author.

## Ethics Statement

The animal study was reviewed and approved by Office of Animal Care and Institutional Biosafety Committee at UIC.

## Author Contributions

SC performed and calculated the echocardiographic, histology, and HOP experiments. CW, DR, and AB performed and analyzed the biochemical experiments. JS performed and analyzed the skinned fiber experiments. EK provided the PLNKO mouse model and edited the manuscript. JT provided the TnT-R92Q mouse model. BW and RS were involved in designing the experiments, writing the manuscript, and financial support. CW contributed also to the financial support. PV was involved in the preparation and review of the manuscript.

## Conflict of Interest

The authors declare that the research was conducted in the absence of any commercial or financial relationships that could be construed as a potential conflict of interest.
